# Influence of surface geometry on the culture of human cell lines: A comparative study using flat, round-bottom and v-shaped 96 well plates

**DOI:** 10.1371/journal.pone.0186799

**Published:** 2017-10-30

**Authors:** Sara Shafaie, Victoria Hutter, Marc B. Brown, Michael T. Cook, David Y. S. Chau

**Affiliations:** 1 Research Centre in Topical Drug Delivery and Toxicology, Department of Pharmacy, Pharmacology and Postgraduate Medicine, School of Life and Medical Sciences, University of Hertfordshire, College Lane, Hatfield, United Kingdom; 2 MedPharm Ltd, Unit 3 Chancellor Court, Surrey, Guildford, United Kingdom; University of Reading, UNITED KINGDOM

## Abstract

*In vitro* cell based models have been invaluable tools for studying cell behaviour and for investigating drug disposition, toxicity and potential adverse effects of administered drugs. Within this drug discovery pipeline, the ability to assess and prioritise candidate compounds as soon as possible offers a distinct advantage. However, the ability to apply this approach to a cell culture study is limited by the need to provide an accurate, *in vitro*-like, microenvironment in conjunction with a low cost and high-throughput screening (HTS) methodology. Although the geometry and/or alignment of cells has been reported to have a profound influence on cell growth and differentiation, only a handful of studies have directly compared the growth of a single cell line on different shaped multiwell plates the most commonly used substrate for HTS, *in vitro*, studies. Herein, the impact of various surface geometries (flat, round and v-shaped 96 well plates), as well as fixed volume growth media and fixed growth surface area have been investigated on the characteristics of three commonly used human cell lines in biopharmaceutical research and development, namely ARPE-19 (retinal epithelial), A549 (alveolar epithelial) and Malme-3M (dermal fibroblastic) cells. The effect of the surface curvature on cells was characterised using a combination of a metabolic activity assay (CellTiter AQ/MTS), LDH release profiles (CytoTox ONE) and absolute cell counts (Guava ViaCount), respectively. In addition, cell differentiation and expression of specific marker proteins were determined using flow cytometry. These *in vitro* results confirmed that surface topography had a significant effect (p < 0.05) on cell activity and morphology. However, although specific marker proteins were expressed on day 1 and 5 of the experiment, no significant differences were seen between the different plate geometries (p < 0.05) at the later time point. Accordingly, these results highlight the impact of substrate geometry on the culture of a cell line and the influence it has on the cells’ correct growth and differentiation characteristics. As such, these results provide important implications in many aspects of cell biology the development of a HTS, *in vitro*, cell based systems to further investigate different aspects of toxicity testing and drug delivery.

## Introduction

*In vitro* cell culture models provide simple, fast and cost-effective tools for biological cell research and help to minimise the exploitation of animal testing [1]. Considerations are required to address the balance between using more complete *in vivo*-like models and the ability to generate a high-throughput screening (HTS) methodology to expedite a typical drug discovery pipeline process. Development of *in vitro* experimental models that closely mimic the *in vivo* microenvironment of the native organ and provide accurate information about *in vivo* biological processes is one of the most challenging aspects of current cell culture research. Traditional long-standing two-dimensional (2D) cell culture models are based on the growth of specific cells on flat and rigid culture substrates/scaffolds within a controlled laboratory environment. These cells are themselves classified into three distinct groups namely, (i) adherent cells which must attach to a solid substrate during culture, (ii) suspension-based cells which are cultured as “floating units” within the culture medium [2], and (iii) cells that exhibit a mixed adherent-suspension characteristic. During an established growth profile of adherent cells, the cultured monolayer is typically comprised of a bulk of proliferating cells with necrotic, unhealthy cells detaching from the culture surface and settling in the surrounding medium. Concurrently, healthy cells in such growth environments maintain their supply of essential nutrients and growth factors through regular replacement of fresh culture medium. The biggest disadvantage of such culture systems is that it does not fully replicate the microenvironment experienced *in vivo* where cells grow within a complex three-dimensional (3D) matrix and, as the 3D structure impacts biological processes from the molecular level (i.e. gene and protein synthesis, and biomolecular gradients) [3] to the proliferation, differentiation and apoptotic nature of the cells, consideration of this key factor must be sought [4].

While continued development of 2D *in vitro* models has been of fundamental importance over the past century for its ease of use, developments within the more appropriate 3D cultures have highlighted some of the fundamental drawbacks associated with the 2D flat monolayers [2]. As such, the growing body of evidence suggests that 3D cell culture models more accurately represent the actual microenvironment where cells reside in native tissues [2]. For instance, in the simplest description, there is only one surface to which cells can adhere due to the innate geometry of a culture substrate. This naturally forces one-sided attachment of the cells and limits any opportunity for cellular contact on the opposite side resulting in a default apical-basal polarity and consequently changes in cell shape and cellular function [5]. Even at the physiological level, Huang and colleagues reported that growth of cells on a 2D surface results in unnaturally flattened and more stretched cells than normally appear *in vivo* [6]. In addition, growing cancer cells on a 3D environment can reveal a more accurate drug response prediction [7] and differential proliferation rate [8]. Previous research also reported that primary mouse mammary luminal epithelial cells maintained a higher proliferation rate on a 3D basement membrane matrix compared to a 2D environment [9]. Furthermore, Lee and colleagues reported different protein expression and sensitivity to chemotherapeutic agents for epithelial ovarian cancer cells cultured on a 3D microenvironment compared with 2D models [10]. Although emphasis over the years has been directed to creating the “ideal” 3D environment which is frequently addressed by using a variety of complicated structured materials, such as gels, solid matrices and custom proprietary materials, difficulties and limitations exist in respect of the, ease of use, biocompatibility and ability to scale-up into an industrially viable process of this model.

A simple methodology to address the appropriateness of a 3D environment of an *in vitro* cell culture model, without using an additional scaffold, is to culture cells on an appropriately shaped culture substrate i.e. based on curvature similar to the native cell microenvironment. In the context of scale-up and HTS, the use of commercially available well plates would be advantageous by allowing replication due to standardisation/quality assurance during their manufacture as well as consideration for cost-effectiveness and technology transfer. In this paper, the effect of (i) growth support topography and (ii) growth culture conditions- in terms of surface area and volume of culture medium available to the cultured cell lines were assessed. Characteristics including cell viability, mitochondrial activity and the cells’ functional characteristics/differentiation-capacity were investigated *in vitro* using a range of biochemical assays and surface marker expression/flow cytometry. Three different cell lines were used within this study and include (i) the immortalised adult human retinal pigment epithelial cells, ARPE-19. These cells reside in a curved microenvironment *in vivo* with a radius curvature of ~12 mm—although exact dimensions can vary considerably based on a number of demographical factors and/or diseased states [11, 12]. ARPE-19 cells are found as a highly pigmented monolayer of cells between Bruch’s membrane and the photoreceptor layer of the neural retina, with individual cells retaining a polygonal/hexagonal shape that are aligned perpendicular to the underlying membrane [13], (ii) the malignant human melanoma skin cell line, Malme-3M, typical residing as a flat microenvironment and, (iii) the human type II alveolar epithelial cell line, A549, grown on the microcurvature of the alveolar surface with a mean alveolar radius curvature of approximately 70 μm [14]. Developing *in vitro* models that consider a cell’s unique *in vivo* growth environment- being more representative of the native tissue- will consequently enhance reliability of cell-based assay findings in basic and clinical research. Importantly, the ability to potentially develop a low cost and standardised HTS drug screening platform in conjunction with enhanced testing accuracy and sensitivity would make a significant contribution to the drug discovery and toxicological research fields.

## Materials and methods

### Materials

The immortalised human retinal pigment epithelial cells, ARPE-19 (ATCC® CRL-2302™), malignant human melanoma skin cell line, Malme-3M (ATCC® HTB-64™), and immortalised human lung adenocarcinoma epithelial cell line, A549 cells (ATCC® CCL185™), were purchased from the ATCC (LGC Standards, Middlesex, UK). Dulbecco's Phosphate Buffered Saline (PBS, pH 7.45), Dulbecco's modified Eagle’s medium (DMEM), Ham's F12 medium, foetal bovine serum (FBS), 2 mM L-glutamine, 100 IU/ml penicillin-100 μg/ml streptomycin solution, 0.25% v/v trypsin-EDTA, Giemsa and May-Grunwald stains, Corning® Costar® flat, round and v-shaped 96 well cell culture plates were obtained from Sigma-Aldrich (Poole, Dorset, UK). Primary mouse monoclonal antibodies for ARPE-19 cell line; anti-CRALBP (ab15051), anti-ARPE65 (ab78036); for A549 cell line; anti-CD74 (ab9514); and for Malme-3M cell line; anti-HLA-A2 (ab74674); were purchased from Abcam (Milton, Cambridgeshire, UK). A secondary antibody, FITC-labelled goat anti-mouse IgG (F0257) was obtained from Sigma-Aldrich (Poole, Dorset, UK). All other chemicals were purchased from Sigma-Aldrich unless otherwise stated. Corning® Costar® well plates (flat: CLS3595, round bottom: CLS3799; v-shaped: CLS3894) were also purchased from Sigma-Aldrich. Sterile preparation of stock solutions and chemicals were performed either by filtration through a 0.22 μm Whatmann sterile filter and/or autoclaving at 121°C at 1 bar for 1 h.

### Methods

#### Human ARPE-19, A549, and Malme-3M cell culture

ARPE-19 cells were cultured using DMEM/Ham's F12 medium supplemented with 10% v/v FBS, 2 mM L-glutamine and 100 IU/ml penicillin-100 μg/ml streptomycin solution. In contrast, the A549 and Malme-3M cells were cultured using DMEM supplemented with 10% v/v FBS, 2 mM L-glutamine and 100 IU/ml penicillin-100 μg/ml streptomycin solution. For routine maintenance, cells were grown in T-75 tissue culture flasks and kept in a humidified-atmosphere incubator at 37°C and 5% CO₂. When cell confluency reached 85% (approximately every 2–3 days), the cells were detached using a standardised trypsinisation method with 0.25% v/v trypsin-EDTA in PBS.

#### Calculation of surface area for cell growth

Consideration to ensure that both fixed surface area (FSA) and fixed volume (FV) during culture needs to be maintained within the appropriate experimental set-up. As such, the following calculations demonstrate that adding 100 μl of cell suspension to a flat well plate provides a surface area of 94.6 mm^2^ well for the cultured cells to grow. From this finding, the subsequent volumes of growth medium to achieve the same growth surface area for cells on round and v-bottom plates were calculated; 117 μl for round well plates and 123 μl for v-shaped well plates. It must be noted that for all the Corning well plates, the entire plate and lid (i.e. total exposed surface area) is prepared and treated by the corona discharge process (information provided by Corning Life Sciences, UK).

As summarised in Table 1, a flat well plate can be visualised as being cylindrical structure of known (circular) diameter; a round well represented as a cylinder on the top and a semi-hemisphere on the bottom, whereas a v-shaped well can be divided into a cylinder on the top and a cone on the bottom.

**Table 1:** Calculation of surface area (SA) based on 100 μl cell suspension volume (V) on flat well plates.

Well plate	Circle	Cylinder	Total	Calculating total SA
**Flat**	1: A = πr^2^ Dimension: r = 3.2mm	2: A = 2πrh 3: V = πr^2^h Dimension: r = 3.2mm	4: A_total_ = πr^2^+2πrh	By substituting 100 μl as cell suspension volume in equation (3), ‘h’ was calculated and replaced in equation (4) to calculate the total surface area equivalent to 100 μl cell suspension in a flat well plate
**Well plate**	Semi-hemisphere	Cylinder	Total	Calculating total SA
**Round-bottom**	5: A = 1/2*4πr^2^ 6: V = 2/3 πr^3^ Dimension: r = 3.2mm	7: A = 2πrh 8: V = πr^2^h Dimension: r = 3.2mm	9: A_total_ = 2πrh+1/2*4πr^2^	By substituting total surface area from equation (4) into equation (9), ‘h’ was calculated and replaced in equations (6) and (8) to calculate the total volume equivalent to 94.6 mm^2^ surface area
**Well plate**	Cone	Cylinder	Total	Calculating total SA
**v-shaped**	10: A = πrS 11: V = 1/3 πr^2^h Dimension: r = 3.2mm S = 3.67 mm h = 1.8mm	12: A = 2πrh 13: V = πr^2^h Dimension: r = 3.2mm	14: A_total_ = 2πrh+πrS	By substituting total surface area from equation (4) into equation (14), ‘h’ was calculated and replaced in equations (11) and (13) to calculate the total volume equivalent to 94.6 mm^2^ surface area

#### Cell mitochondrial activity

To investigate the effect of surface geometry on the activity of ARPE-19, A549 and Malme-3M cells, the CellTiter96 AQueous One Solution Cell Proliferation assay kit (Promega, Southampton, UK) was used according to the manufacturer’s instruction. In brief, cells were plated onto flat, round and v-shaped 96 well plates at a density of 1 x 10^4^ cells per well. Exact volumes per condition are summarised as above: 100 μl/well for FV; whereas for FSA, the volume of media varies depending on the well plate used; flat: 100 μl, round: 117 μl and v-shaped 123 μl/well.

At 24 h intervals, a 10 μl aliquot of the CellTiter One reagent was added to each well of the appropriate 96-well plate that contained the cell samples in 50 μl of culture medium (1:5 dilution). Samples were placed thereafter into a humidified-atmosphere incubator at 37°C, 5% v/v CO₂ for 180 min. The absorbance was read at 492 nm using the Multiskan Ascent 96/384 plate reader (MTX Lab Systems, Virginia, USA). Corresponding media for each cell line was used as a background control.

#### Cell cytotoxicity

The CytoTox-ONE™ Homogeneous Membrane Integrity assay kit (Promega, Southampton, UK) was used to measure the amount of lactate dehydrogenase (LDH) released from the cells. ARPE-19, A549 and Malme-3M cells were plated in 96 flat, round and v-shaped well plates at a density of 1 x 10^4^ cells per well. At 24 h intervals, 50 μl of cell supernatant from each well was transferred to its corresponding well on a new black 96 well plate, followed by the addition of 50 μl of CytoTox-ONE™ Reagent (1:1 ratio). Cells were then incubated in the dark for 10 minutes at room temperature (~19°C) before 25 μl of stop solution was added. Fluorescence was measured immediately after addition of stop solution at 525 nm excitation wavelength and 580–680 nm emission wavelengths using a GloMax-Multi^+^ microplate reader (Promega, Southampton, UK). The fluorescence of each background control was subtracted from the fluorescence of the corresponding experimental well to obtain the corrected fluorescent reading.

#### Cell viability

Flow cytometry in conjunction with the Guava ViaCount Assay kit (Merck Millipore, Watford, UK) can provide an absolute cell count (1:1) as well as viability characteristics of a cell sample by distinguishing between viable and non-viable cells based on the differential permeability of DNA-binding dyes in the ViaCount reagent. ARPE-19, A549 and Malme-3M cells were seeded on flat, round bottomed and v-shaped 96 well plates at a density of 1 x 10^4^ cells/well and were maintained at 37°C, 5% v/v CO_2_ in a humidified incubator. Cells were harvested using a standard trypsinisation technique followed by deactivation with 100 μl of fresh media at 24 h intervals. From each well, 100 μl of cell suspension was transferred to a new well plate and 100 μl of Viacount reagent was then added to the well. Cells were incubated in the dark for 5 minutes at room temperature (~19°C). After staining, viability data was acquired using the Guava 8HT flow cytometer with dual blue (488 nm) and red (635 nm) excitation lasers and a total acquisition of 3000 events per sample. Experiments were conducted in triplicate and data was analysed using the in-built GuavaSoft™ Viacount 2.7 software.

#### Attachment and spreading assay

Cells were seeded at 1 x 10^4^ cells per well on the flat, round and v-shaped 96 well plates. At the corresponding time-points, the medium was removed and cells washed twice with 100 μl of PBS solution, pH 7.4. Following that, cells were fixed and permeabilised for 15 min at room temperature (~19°C) with 100 μl of 3.7% w/v paraformaldehyde in PBS and 0.1% v/v Triton X-100, pH 7.4. After which, cells were first treated with 100 μl of 0.25% w/v May-Grunwald stain, in methanol, for 15 min at room temperature (~19°C) and then nuclear staining was achieved by adding 100 μl of 0.4% w/v Giemsa stain, in methanol (diluted 1:20 with distilled water). Following a 20 minute incubation, at room temperature (~19°C), the stains were removed and the sample washed once with distilled water and left to air dry. Extent of attachment and spreading characteristics of the cells was visually assessed by using X4 and X10 objective magnifications using GX CAM digital camera and Meiji EMT microscope (Meiji Techno, Somerset, UK).

#### Expression of cell specific markers using flow cytometry

Expression of cell surface markers for ARPE-19 (RPE-65 and CRALBP), A549 (CD74) and Malme-3M (HLA-A2) cell lines was determined by the addition of monoclonal antibodies with specific affinity for the investigated surface markers. ARPE-19, A549 and Malme-3M cells were seeded at 1x 10^4^ cells per well on each different well type before being fixed using 3.7% v/v formaldehyde in PBS on days 1 and 5 post seeding. ARPE-19 cells were additionally permeabilised with 0.1% v/v Triton X-100, for 10 min, due to the cytoplasmic location of the cell markers. Thereafter, samples were blocked with 3% w/v BSA, in PBS, for 10 min at room temperature (~19°C) before being incubated with 2 μl of cell specific primary antibodies for 45 min at room temperature (~19°C). Following a wash step with PBS, samples were then incubated with a secondary antibody, FITC-labelled goat anti-mouse IgG (1:500) in 1% w/v BSA in PBS, at 4°C for 45min. Samples were then washed with 100 μl of 1% w/v BSA in PBS, centrifuged at 1200 rpm for 5 min. An untreated sample (unstained cells) and the secondary antibody control (cells stained with secondary antibody) were included in each analysis to address auto-fluorescence and non-specific binding, respectively. For data analysis, each sample population was gated to only include healthy cells of based on their forward scatter (cell size) and side scatter (cell granularity) profiles. A total of 3000 events were collected for each sample. Raw data was collected using Guava EasyCyte™ 8HT Flow Cytometer (Merck Millipore, Watford, Hertfordshire, UK) with dual blue (488 nm) and red (635 nm) excitation lasers. Data were analysed using GuavaSoft™ 2.7 software and InCyte™ analyser. The absolute median fluorescent intensity (MFI) value for each marker was determined.

#### Statistical analysis

Data are presented as mean ± SD (standard deviation), and compared using two-way ANOVA with Bonferroni post-hoc test. Statistical significance between the control group (flat well plate) and the experimental groups (round and v-shaped plates) is indicated with (*) which represents a *p* < 0.05, (**) which represents a *p* < 0.01, and (***) which represents a *p* < 0.001.

## Results

### Comparison of the effect of growth surface topography and growth media volume on cell activity, viability and LDH release of ARPE-19 cells

**Fig 1:**
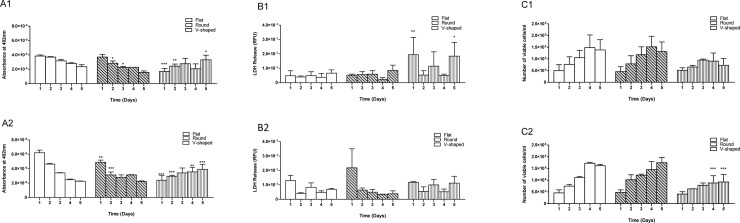
Comparing cell activity, LDH release and viability of ARPE-19 cells cultured on flat, round and v-shaped well plates at 1 x 10^4^ cells per well density over 5 days and at fixed growth volume and fixed growth surface area. Group A: Mitochondrial activity of ARPE-19 cells cultured at 1 x 10^4^ cells per well density; A1) cell activity cultured on flat, round and v-shaped well plates at FV and relative to flat well plates; A2) cell activity cultured on flat, round and v-shaped well plates at FSA and relative to flat well plates. Group B: LDH release measurements of RPE-19 cells cultured at 1 x 10^4^ cells per well density; B1) LDH release on flat, round and v-shaped plates at FV and relative to flat; B2) LDH release on flat, round and v-shaped plates and relative to flat at FSA. Group C: cell viability/ml of ARPE-19 cells cultured at 1 x 10^4^ cells per well density; C1) flat, round and v-shaped cell viability relative to flat well plates at FV; C2) flat, round and v-shaped cell viability relative to flat well plates at FSA. Data are represented as mean + SD of three independent repeats (n = 3). Any significant difference in results is shown as *p* ≤ 0.05*, *p* ≤ 0.01**, *p* ≤ 0.001***.

Over a 5-day experiment, surface geometry of cell culture well plates had a significant effect on cell activity (FV and FSA: *p* ≤ 0.001), death (FV: ns and FSA: *p* ≤ 0.01) and viability (FV: *p* ≤ 0.01 and FSA: *p* ≤ 0.001) of cultured ARPE-19 cells, Fig 1. Cells on all three different well plates followed a standard bell-shaped growth curve for both FV and FSA, reaching highest mitochondrial activity on day 4 post seeding. Despite a decline in number of viable cells on v-shaped wells after 3 days in culture no significant difference (*p* > 0.05) was observed for LDH release. Cell activity was significantly lower (*p* < 0.05) for cells cultured on v-shaped wells at both FV and FSA in comparison with flat and round well plates. However fewer cells were present when cultured on the v-shaped wells. In addition, two-way ANOVA statistical comparison between mitochondrial activity of ARPE-19 cells on round and v-shaped plates and at the flat FV and FSA growth conditions also demonstrated that cells cultured with FSA produced significantly greater absorbance values compared to those cultured at fixed growth volume; round plates at FSA: day 4 *p* ≤ 0.001 and day 5, and v-shaped plates at FSA: day 4 *p* ≤ 0.05.

### Comparison of the effect of growth surface topography and growth media volume on cell activity, viability and LDH release of A549 cells

**Fig 2:**
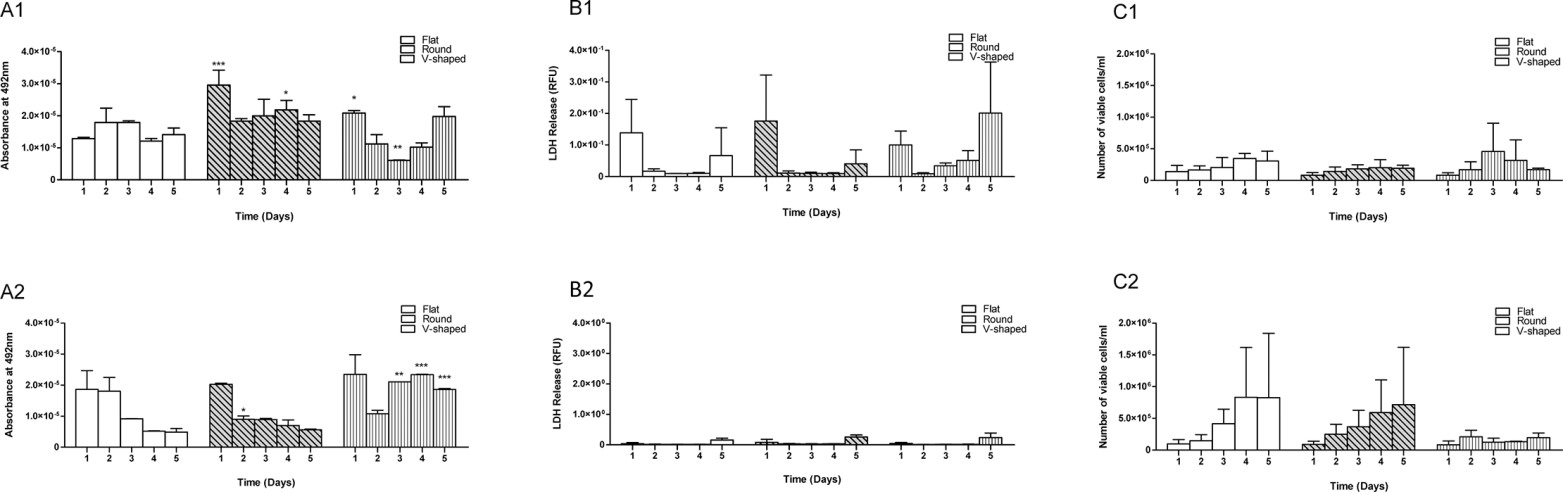
Comparing cell activity, LDH release and viability of A549 cells cultured on flat, round and v-shaped well plates at 1 x 10^4^ cells per well density over 5 days and at fixed growth volume and fixed growth surface area. Group A: Mitochondrial activity of A549 cells cultured at 1 x 10^4^ cells per well density, A1) cell activity cultured on flat, round and v-shaped well plates and relative to flat at FV; A2) cell activity cultured on flat, round and v-shaped well plates and relative to flat at FSA. Group B: LDH release of A549 cells cultured at 1 x 10^4^ cells per well density, B1) LDH release on flat, round and v-shaped plates and relative to flat at FV; B2) LDH release on flat, round and v-shaped plates and relative to flat at FSA. Group C: cell viability/ml of A549 cells cultured at 1 x 10^4^ cells per well density; C1) flat, round and v-shaped cell viability relative to flat well plates at FV; C2) flat, round and v-shaped cell viability relative to flat well plates at FSA. Data are represented as mean + SD of three independent repeats (n = 3). Any significant difference in results is shown as *p* ≤ 0.05*, *p* ≤ 0.01** and is relative to flat plates.

Based on the results from a two-way ANOVA analysis, surface topography had a significant effect on the mitochondrial activity of A549 cells at FV and FSA (*p* ≤ 0.01), a significant effect on the LDH release profile at FV (*p* ≤ 0.05) but demonstrated no significant effect (*p* > 0.05) on cell viability at either the FV or FSA growth conditions, Fig 2. For all growth conditions, a plateau was reached after 5 days in culture. There was no significant difference between the three well plate geometries used and mitochondrial activity at FV (*p* > 0.05). In contrast, at FSA, cells on flat well plates had a significantly greater mitochondrial activity on days 3 and 4 (*p* ≤ 0.01) in comparison with v-shaped well plates. In terms of LDH release (i.e. characteristics of cell death), there were no significant difference between the cultured cells on the three different well plates at FV or FSA (*p* > 0.05). Two-way ANOVA statistical analysis confirmed that for each specific well plate, there was no significant difference in mitochondrial activity, cell death and viability between A549 cells at FV or FSA despite a lower number of cells on v-shaped wells in comparison with flat and round well plates.

### Comparison of the effect of growth surface topography and growth media volume on cell activity, viability and LDH release of Malme-3M cells

**Fig 3:**
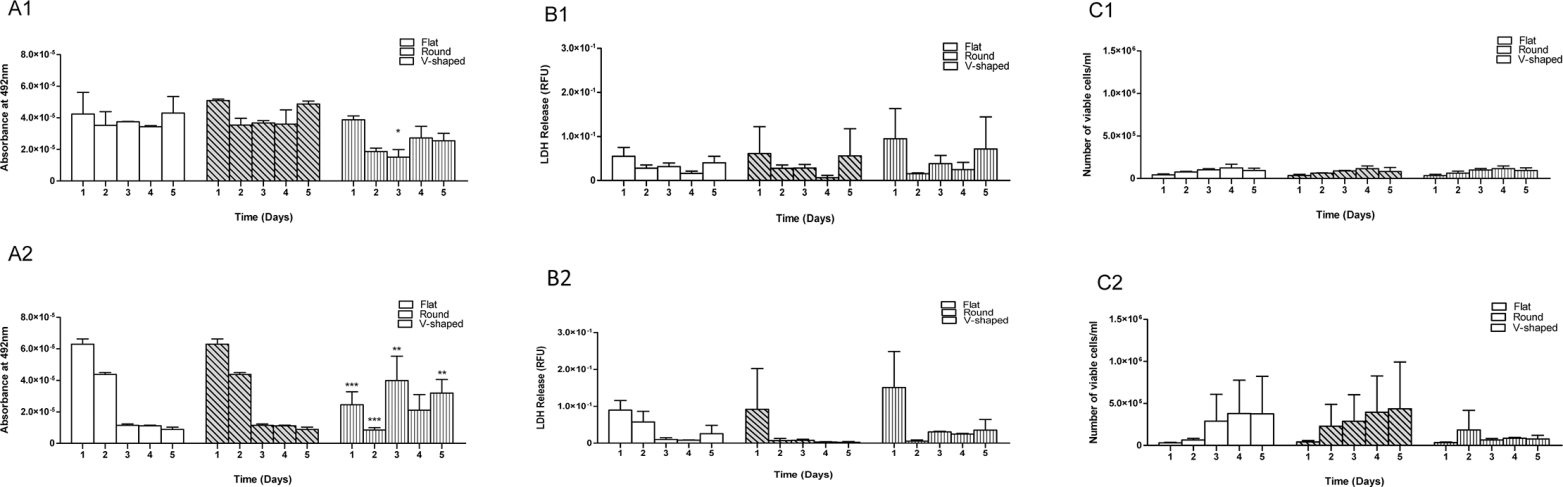
Comparing cell activity, LDH release and viability of Malme-3M cells cultured on flat, round and V-shaped well plates at 10^4^ cells/well density over 5 days and at fixed growth volume and fixed growth surface area. Group A: Mitochondrial activity of Malme-3M cells cultured at 10^4^ cells/well density, A1) cell activity cultured on round and V-shaped well plates and relative to flat at FV; A2) cell activity cultured on round and V-shaped well plates and relative to flat at FSA. Group B: LDH release of Malme-3M cells cultured at 10^4^ cells/well density, B1) LDH release on round and V-shaped plates and relative to flat at FV; B2) LDH release on round and V-shaped plates and relative to flat at FSA. Group C: cell viability/ml of Malme-3M cells cultured at 10^4^ cells/well density l, C1) round and V-shaped cell viability relative to flat well plates at FV; C2) round and V-shaped cell viability relative to flat well plates at FSA. Data are represented as mean + SD of three independent repeats (n = 3). Any significant difference in results is shown as *p* ≤ 0.05*, *p* ≤ 0.01**, *p* ≤ 0.001*** and is relative to flat plates.

Results from the two-way ANOVA analysis confirmed that surface topography had a significant effect (*p* ≤ 0.001) on the mitochondrial activity of the Malme-3M cells cultured on the three different well plates, at both the FSA and FV environments. In contrast, despite lower number of cells on v-shaped well plates and at FSA, no significant differences (*p* < 0.05) were observed for the LDH release profile or cell viability characteristics when cultured in the corresponding well plates, as seen in Fig 3. Absorbance values for cells on flat well plates were significantly higher in comparison with that of v-shaped well plates; FV day 2 *p* ≤ 0.05, day 3 *p* ≤ 0.001, day 5 *p* ≤ 0.01, and FSA: day 1 *p* ≤ 0.05, day 4 *p* ≤ 0.001. As observed for ARPE-19 cells, the Malme-3M cells also displayed a characteristic growth curve for all three well plates and at both growth conditions with highest mitochondrial activity and absorbance reading observed on day 4. Two-way ANOVA statistical analysis confirmed that for each specific well plate, there was no significant difference in cell mitochondrial activity between FV and FSA growth conditions.

Cell toxicity results also confirmed no significant difference (*p* < 0.05) in cell death and LDH release between cells cultured on flat and round bottomed well plates for the three cell lines. Further confirmation of this behaviour can be noted in the cell viability results as seen in Figs 1–3.

### Effect of growth surface topography and growth media volume on attachment and spreading of ARPE-19, A549 and Malme-3M cells

**Fig 4:**
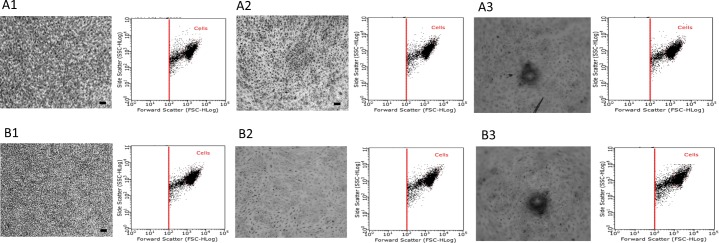
Attachment and spreading of ARPE-19 cells on different surface topographies and at different growth conditions. ARPE-19 cultured at 1 x 10^4^ cells per well density on flat, round and v-shaped well plates over 8 days and at fixed growth volume and fixed growth surface. Group A and B represent cells at FV and FSA respectively and images 1, 2 and 3 represent flat, round and v-shaped well plates respectively. Samples were photographed using GX CAM digital camera at X 4 magnification (100 μm = scale bar). Each images is accompanied by analysis of the cells in the dot plot display mode of forward scatter (FCS) versus side scatter (SSC) on a logarithmic scale, and the core population of the cells is surrounded by a gate.

**Fig 5:**
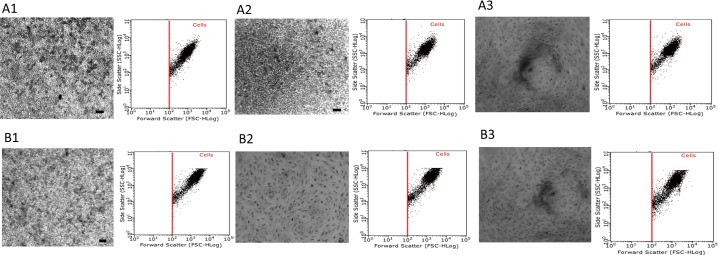
Attachment and spreading of A549 cells on different surface topographies and at different growth conditions. A549 cultured at 1 x 10^4^ cells per well density on flat, round and v-shaped well plates over 8 days and at fixed growth volume and fixed growth surface. Group A and B represent cells at FV and FSA respectively and images 1, 2 and 3 represent flat, round and v-shaped well plates respectively. Samples were photographed using GX CAM digital camera at X 4 magnification (100 μm = scale bar). Each images is accompanied by analysis of the cells in the dot plot display mode of forward scatter (FCS) versus side scatter (SSC) on a logarithmic scale, and the core population of the cells is surrounded by a gate.

**Fig 6:**
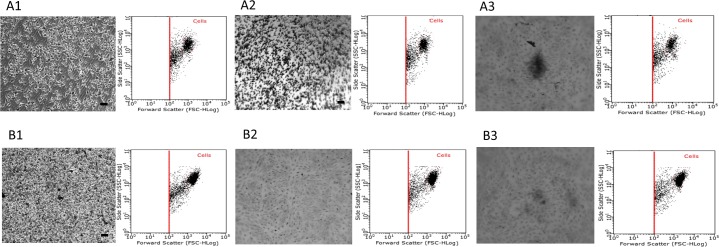
Attachment and spreading of Malme-3M cells on different surface topographies and at different growth conditions. Malme-3M cultured at 1 x 10^4^ cells per well density on flat, round and v-shaped well plates over 8 days and at fixed growth volume and fixed growth surface. Group A and B represent cells at FV and FSA respectively and images 1, 2 and 3 represent flat, round and v-shaped well plates respectively. Samples were photographed using GX CAM digital camera at X 4 magnification (100 μm = scale bar). Each images is accompanied by analysis of the cells in the dot plot display mode of forward scatter (FCS) versus side scatter (SSC) on a logarithmic scale, and the core population of the cells is surrounded by a gate.

Images taken after dual staining with Giemsa and May-Grunwald confirmed that cells showed normal attachment and spreading onto flat and round well plates. However, images taken from v-shaped plates suggest that the *in situ* localisation of the cells were affected by its innate topography: cells appearing to aggregate in the centre of the well plate and also demonstrate a more elongated morphology as seen in Figs 4–6. This is also confirmed using the flow cytometry profiles (inserts) of each experimental condition- cells characterised by their forward scatter (i.e. size) and side scatter (i.e. granularity).

### Cell marker expression for ARPE-65, CRALBP, CD74 and HLA-A2

The expression of the cell specific protein markers for each cell line was evaluated between the different culture environments i.e. growth surfaces and at FV and FSA conditions using flow cytometry on days 1 and 5 of the experiment. Results demonstrated that ARPE-19, A549 and Malme-3M cells cultured on flat, round and v-shaped well plates consecutively expressed cell specific protein markers at both fixed growth volume and fixed growth surface area. Two-way ANOVA analysis confirmed that for each cell line, there is no significant difference in MFI of expressed cell specific markers cultured on different cell growth surfaces and different growth conditions, hence surface topography and culture conditions did not adversely affect the expression of RPE-65 and CRALBP (retinal), CD74 (alveolar), and HLA-A2 (skin) specific markers.

## Discussion

Limitations associated with *in vivo* models including ethical concerns, cost, and anatomical and physiological differences among interspecies have led to development and validation of various alternative *in vitro* models derived from animal and human primary and immortalised cells [15]. Standard cell culture models are widely used to understand the biological, chemical and molecular cues of living cells [16]. However, a number of these simple systems are based on single cell monolayers cultured on a 2D culture scaffold and do not take into account the actual microenvironment in which they reside within the native tissue [15]. Although investigation of cell proliferation, differentiation, and function *in vitro* within these monolayer systems has led to many significant findings, it is often reported that changing the growing environment of cultured cells can considerably change function and capacity for cell growth and differentiation [17]. The current research direction within the cell culture field is now considering the integration of 3D microenvironment that allows structured cell growth in the appropriate dimensions and evidence suggests that these multi-cellular 3D structures significantly differ to the conventional 2D monolayer cultures [18–21]. However, the biggest disadvantage within these systems is that they rely on the use of 3D scaffolds. The majority of these are complex solid structures and/or custom synthesised gels which has, thus far, limited the translation to high-throughput processing and reproducibility. Accordingly, to the best of our knowledge, no study currently exists that compares the growth characteristics of cells on different surface geometries in a reproducible and HTS-ready environment; essentially comparing a cell line cultured on a flat, round or v-shaped 96 well plates. Such an observation could have major implications in the development of complex 3D models for regenerative medicine and more sensitive *in vitro* screening cell-based models. In support of this hypothesis, although a number of *in vitro* ocular models currently exist for drug toxicity, irritation and absorption studies [15, 22, 23], none have directly considered the curvature and geometry of the *in vivo* eye, or investigated the effect of curved growth scaffolds on cell characteristics and the associated cell-cell interactions. In this work, the initial stages of developing a HTP-ready, *in vitro* ocular model that will evolve towards a 3D *in vitro* model by investigating cell growth on curved culture scaffolds mimicking ARPE-19 growth microenvironment *in vivo* is reported. In addition, the cell characteristics of two additional cell lines, A549 and Malme-3M cells, to mimicking the lung and skin, respectively, were also investigated.

Accordingly, for these three cell lines, cell activity is only adversely affected by v-shaped supports and that there is no significant difference (*p* > 0.05) between the metabolic activity of the cells cultured on the round bottom and on the flat well plates. A simple explanation for this observation can be, in part, related to the discrete surface geometry of the v-shaped well in comparison with cells’ native tissue *in vivo*. Upon culturing on v-shaped well plates, cells naturally localise (i.e. gravitational) towards the middle and at the bottom of the plate. This, in turn, limits the possibility of mass transfer in respect to their access to nutrients and oxygen resulting in an increase in cell death with decreased cell activity.

Surface topography is critical to guide cellular behaviours such as adhesion, spreading and migration [24, 25]. As cell adhesion on scaffold surfaces is a primary step to guide cellular function and tissue generation [26, 27], it was important to investigate the effect of surface geometry on the attachment and spreading of cultured cells. As seen inFigs 4–6, all three cell lines attached and spread throughout the growth surface area available on the flat well plates and retained their structural and morphological characteristics of cells *in vivo*. Cell adhesion on round well plates followed a similar behaviour but were not able to fully cover and attach to all the growth surface area available- represented by the absence of cells around the outer edges of the wells were (Fig 5, A2). In contrast, on the v-shaped well plates, the cells were mainly localised in the middle and at the bottom of the cone. These observed results are in agreement with a similar study performed by Kim and colleagues who investigated the influence of concave and convex architectures on the response of human epithelial cells and reported that it was possible to selectively influence either cell adhesion or morphology via surface topography [27]. In addition, Ghibaudo *et al*, studied the role of substrate topography in cell adhesion and migration. They showed that substrates with various well-defined geometries affect the adhesion and migration of fibroblasts [28]. Similarly, Wang *et al*, confirmed that cell shape, migration, and adhesion can be influenced by surface topography of a growth substrate [29].

**Fig 7:**
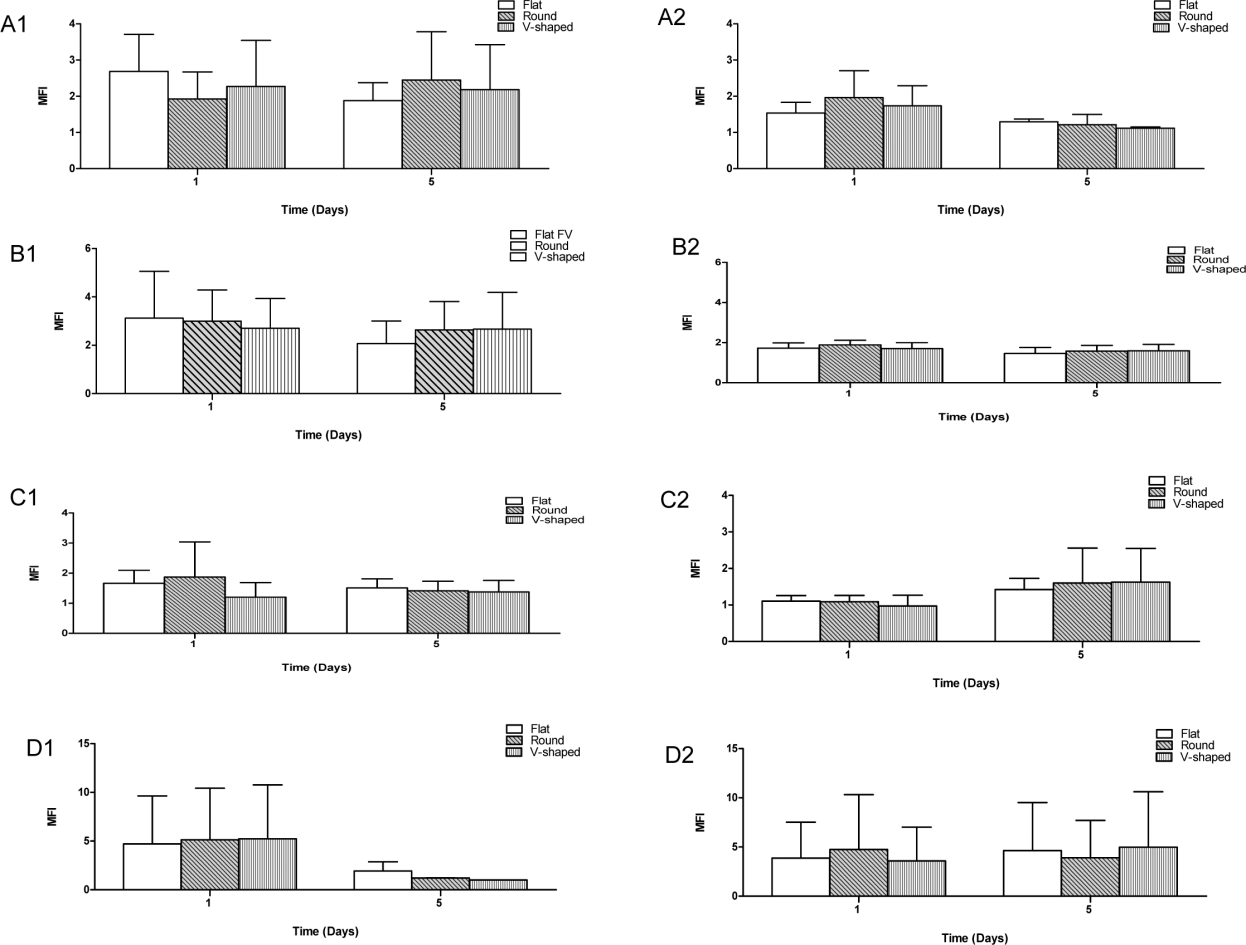
Cell specific marker expression for RPE-65, CRALBP, CD74 and HLA-A2 using flow cytometry via indirect staining. Median Fluorescent Intensity (MFI) data for cell specific marker expression of ARPE-19, A549, and Malme-3M cell lines cultured at 1 x 10^4^ cells per well density on flat, round and v-shaped well plates over 5 days and at fixed growth volume and fixed growth surface area. Samples incubated with 2 μl per well primary antibodies and labelled with goat anti-mouse IgG FITC-tagged secondary antibody (1:500). Data is representative of 3 independent repeats and is shown as mean + SD (n = 3). Group 1: Fixed volume growth condition and Group 2: Fixed surface area growth condition showing cell specific marker expression of A) RPE-65 (ARPE-19 cell line), B) CRALBP (ARPE-19 cell line), C) CD74 (A549 cell line) and D) HLA-A2 (Malme-3M cell line) marker proteins.

As substrate surface topography affects cell morphology, consequently the alteration of cell morphology was reported to affect cell proliferation and phenotype [26] For instance, the commitment of human mesenchymal stem cells (hMSCs) to adipocyte or osteoblast phenotype was regulated by cell shape: the widely spread and flattened hMSCs experienced osteogenesis, whilst round and unspread hMSCs underwent adipogenesis [30]. Furthermore, Folkman *et al*, found that the shape of mammalian cells (i.e. bovine endothelial cells and WI-38 human fetal lung fibroblasts) is tightly coupled to DNA synthesis. Extremely flat shaped cells expressed almost 30 fold more ^3^H-thymidine in comparison with spheroidal conformation cells, proposing much more active DNA synthesis of broadly spread cells [31]. However, as demonstrated by the flow cytometry results (Fig 7), neither surface topography nor growth conditions adversely affected expression of cell specific markers for ARPE-19, A549 and Malme-3M cell lines. There were no significant differences (*p* > 0.05) between the expression levels of RPE65, CRALBP, CD74, and HLA-A2 specific markers within three well plates and between FSA and FV growth conditions at each time point which suggests that the phenotype and functionality of these cells remain unaffected by the culture conditions. It must be noted that reaction of cells to surface topography is both cell type and time dependent [29] and these observations may not be representative of the long-term characteristics of the cells.

## Conclusion

To date, there have been a few *in vitro* models that have presented the ground work for new anatomically realistic cell culture models which can be used to better represent the complex *in vivo* environment. In this paper, we report that there are limited differences in cell characteristics on cells that are cultured on substrates that mimic the curvature present in their native microenvironment in HTS manner. Importantly, no adverse behaviour between the cells when cultured on either flat or round bottom well plates was observed. These results provide the steps towards developing more accurate, simple high throughput, *in vitro* cell systems that represent the native tissue and may be used to further investigate the differences, if any, in toxicity testing and drug delivery applications.
